# Spatiotemporal Dynamics of the 2025–2026 Measles Outbreak in Mexico: Jalisco as the Emerging Epicenter and the Impact of Intensive Immunization Efforts

**DOI:** 10.3390/pathogens15060572

**Published:** 2026-05-27

**Authors:** Elena Sandoval-Pinto, Rosa Cremades, Héctor Raúl Pérez-Gómez, Roberto Carlos Rivera-Ávila, Cesar Augusto Domínguez Barbosa, Lakshmanane Premkumar, Erick Sierra-Díaz, Diana Emilia Martínez-Fernández, José Ángel Regla-Nava

**Affiliations:** 1 Departamento de Biología Celular y Molecular, Centro Universitario de Ciencias Biológicas y Agropecuarias, Universidad de Guadalajara, Zapopan 45200, Mexico; elena.sandovalp@academicos.udg.mx; 2 Departamento de Microbiología y Patología, Centro Universitario de Ciencias de la Salud (CUCS), Universidad de Guadalajara, Guadalajara 44340, Mexico; rosa.cremades@academicos.udg.mx; 3 Secretaría de Salud Jalisco, Guadalajara 44100, Mexico; hector.pgomez@academicos.udg.mx (H.R.P.-G.); roberto.carlos.rivera.avila@gmail.com (R.C.R.-Á.); augustocesar1@hotmail.com (C.A.D.B.); 4 Instituto de Patología Infecciosa y Experimental, Centro Universitario de Ciencias de la Salud (CUCS), Universidad de Guadalajara, Guadalajara 44280, Mexico; 5 Department of Microbiology and Immunology, University of North Carolina, Chapel Hill, NC 27599, USA; prem@med.unc.edu; 6 División de Epidemiología, Unidades Médicas de Alta Especialidad, Hospital de Especialidades, Centro Médico Nacional de Occidente, Guadalajara 44340, Mexico; erksland@gmail.com; 7 Instituto Transdisciplinario de Investigación y Servicios (ITRANS), Universidad de Guadalajara, Zapopan 45150, Mexico; diana.martinez@academicos.udg.mx

**Keywords:** measles outbreak, vaccination coverage, Jalisco (Mexico), epidemiological surveillance, public health response

## Abstract

Measles represents a critical public health challenge due to its high transmissibility and the resurgence of outbreaks of the disease, linked to gaps in vaccination coverage. In this study, we analyze the epidemiological dynamics of the outbreak that began in Mexico in 2025 and focus on Jalisco as the epicenter during the first quarter of 2026. A descriptive, retrospective, ecological time-series analysis was conducted using official epidemiological surveillance data, correlating incidence with immunization strategies. The results reveal that 6681 cases were recorded in Jalisco, with the highest prevalence among adults aged 20 to 49 years (41.96%), which highlights the accumulated gaps in immunity coverage. The public health response, comprising the administration of three million doses, focusing on zero-dose children and vaccination blockades, contributed to a notable decrease in infections by epidemiological week 17 of 2026. It was concluded that the outbreak was controlled through intensive and coordinated intervention. However, the proximity of the 2026 International Federation of Association Football (FIFA) World Cup requires vaccination coverage to be maintained at over 95%, in addition to vigilance, to mitigate the risk of viral reintroduction and to safeguard global health security.

## 1. Introduction

Measles is one of the most contagious viral diseases known to humankind, with a basic reproduction number (R0)—representing the average number of secondary cases generated from a single initial patient—ranging from 12 to 18. It is a systemic disease and is most common in childhood [1]. It is caused by the measles virus (MeV), a negative-sense, non-segmented, single-stranded RNA virus belonging to the genus *Morbillivirus* of the family *Paramyxoviridae* [2]. The virus is mainly spread through the air via microdroplets at short distances, with a 90% probability of infecting potential direct contacts [2,3]. Other transmission mechanisms include airborne aerosols or contact with viral particles on surfaces, which can remain infectious for up to two hours [2,4].

Clinical manifestations appear after a viral incubation period of approximately 10 days, during which the patient is highly contagious, and are characterized by high fever, cough, and Koplik’s spots (small, white, ulcerative lesions on the oral mucosa considered the pathognomonic sign of the disease), followed by a rash, generally at 14 days, and occasionally complications such as pneumonia, encephalitis, subacute sclerosing panencephalitis (SSPE), and death in the most severe cases [3,4,5,6].

Although the host typically develops a robust immune response that facilitates viral clearance and confers lifelong immunity, MeV paradoxically induces transient immunosuppression. The destruction of memory immune cells due to the infection is accompanied by a loss of immunological memory, known as immune amnesia, which is typical of this disease. This immunological impairment significantly increases long-term morbidity and mortality, leaving the individual vulnerable to secondary infections for several years after recovery [5].

To combat this disease, vaccination is recommended for those over 12 months of age. This requires two doses and carries a 97% protection rate [7,8]. In regions where measles is endemic, the first vaccine is usually administered at 9 months, and in cases of outbreaks, it can be administered from 6 months. This vaccine has proven long-term safety, demonstrates high efficacy against all circulating genotypes of the virus, and prevents community transmission thanks to the high coverage rate guaranteed by its two doses [1]. In accordance with International World Health Organization (WHO) guidelines, Mexico administers the vaccine in its triple viral (MMR) presentation—which protects against measles, mumps, and rubella—or double viral (MR) (measles and rubella) presentations, depending on availability [9]. In terms of public health, the success of these vaccination campaigns is measured through the elimination of disease transmission. Technically, a region is considered measles-free once 12 months have lapsed without confirmed local transmission. However, the stability of this achievement depends on extremely high vaccination rates. Because the virus is highly contagious in enclosed spaces or areas with high population density, any gaps in herd immunity can be exploited by imported cases, justifying the international goal of achieving 95% vaccination coverage [10]. A historical benchmark is the elimination program launched by the United States in 1978, which was based on three pillars: high levels of vaccination immunity, strict epidemiological surveillance, and aggressive outbreak control. Through this sustained strategy, the country achieved official measles elimination certification in 2000 [10].

Measles outbreaks disproportionately affect high-risk populations, including pregnant women, immunocompromised individuals, and malnourished children. In patients with cancer or HIV, the virus is associated with a higher incidence of fatal complications, such as pneumonia or encephalitis, even among previously vaccinated groups. Approximately 45% of measles-related deaths occur in the context of nutrient deficiencies [1]. Regarding maternal health, the infection serves as a significant risk factor for adverse perinatal outcomes, increasing rates of fetal loss, low birth weight, and maternal mortality in vulnerable regions [1,10].

Despite these advances, measles has re-emerged globally due to vaccine hesitancy and disruptions to health services inherited from the COVID-19 pandemic. With nearly 400,000 global cases in 2024 and an upward trend at the start of 2025, the severity of the epidemiological situation is reflected in the high hospitalization rates, which may suggest significant underreporting of cases [1]. In this context, a recent study reported that sociodemographic factors and heterogeneity in vaccine coverage contributed significantly to the measles outbreak in Mexico that began in 2025 [11].

Although measles transmission has been documented across various Mexican states, Jalisco has emerged as the epicenter of the national outbreak in 2026. This geographical shift necessitates a localized analysis to understand the drivers of the current surge; therefore, in this study, we characterize the 2025–2026 outbreak in Mexico, prioritizing the spatiotemporal dynamics within Jalisco. We evaluate primary infection sources, case notification trends, and vaccination coverage gaps to identify the factors influencing viral dissemination. By focusing on the epicenter, this work highlights the specific challenges of subnational outbreak control and the public health response within high-burden regions.

## 2. Materials and Methods

A descriptive, retrospective, ecological time-series study was conducted to analyze the incidence of measles and immunization coverage in the state of Jalisco. The study period spanned from 1 January 2025, to epidemiological week 17 of 2026. A chronological analysis was performed for each epidemiological week to determine the number of cases, incidence rate, mortality, and the number of vaccines administered. Both MMR and MR vaccine types were included depending on availability, without distinguishing between the quantities of each. An epidemiological week begins on Sunday and ends on Saturday.

The data were obtained from the National Epidemiological Surveillance System (SINAVE), specifically from the Special Epidemiological Surveillance System for Measles and febrile Exanthematous Diseases of the General Directorate of Epidemiology (DGE), the platform used to register and monitor measles cases [12,13]. The public health surveillance framework adheres to the protocols stablished by Official Mexican Standard NOM-017-22A2-2012 [14]. Records of administered measles vaccine doses were also obtained from the relevant platform of the National Center for Child and Adolescent Health (CENSIA) [15] which operates in accordance with the guidelines established by NOM-036-SSA2-2012 [16]. The information was processed by the General Directorate of Public Health of the Jalisco Ministry of Health (as of 4 May 2026). Data were analyzed with Prism software v9.1.1 (GraphPad Software).

For data analysis, information was collected at the federal and state levels across all Mexican states to establish a national comparative framework, using the state of Jalisco as the primary unit of analysis. The dataset comprised confirmed measles cases, case epidemiological characteristics, and the number of vaccine doses administered, analyzed annually and by epidemiological week.

Weekly incidence and case-fatality rates were compared between Chihuahua and Jalisco using the Wilcoxon signed-rank non-parametric test, considering each epidemiological week as a paired observation. Temporal association between weekly incidence curves was assessed using Spearman’s rank correlation coefficient. Cumulative incidence rate ratios were also calculated to compare the overall disease burden between states.

## 3. Results

### 3.1. Spatiotemporal Evolution: From Northern Outbreak to the Epicenter of Jalisco

In Mexico, the outbreak began on 14 February 2025, with the first case in Oaxaca. It involved a five-year-old girl with no vaccination history who reportedly traveled to Laos, Vietnam, Japan, and the United States. Although this represented the earliest detected imported case, epidemiological evidence suggests that the main outbreak cluster in Mexico may have originated from a separate imported case reported on 20 February in the northern state of Chihuahua. The patient, a nine-year-old boy, had also traveled to the Seminole community in West Texas. This region, characterized by a significant Mennonite population, presents an increased risk of measles transmission due to suboptimal vaccination coverage rooted in cultural and religious beliefs [17]. From these initial imported cases, the virus spread rapidly across the country. To better understand the dynamics of the current measles outbreak, we analyzed measles data in Mexico from 2025 through to epidemiological week 17 of 2026. Within a short period, several measles cases had accumulated in different states in Mexico.

**Figure 1:**
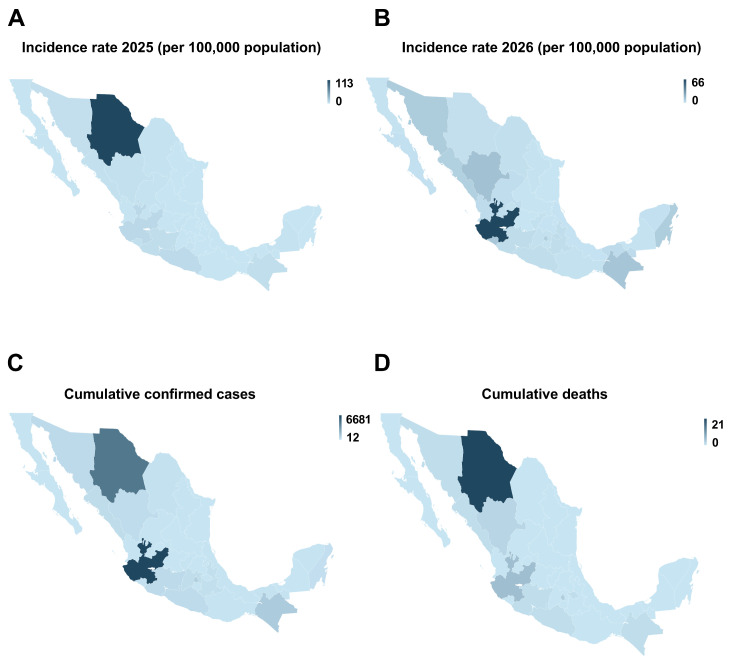
Geographical distribution of measles incidence, cases, and deaths in Mexico, 2025–2026. (**A**) Incidence rate in 2025 (rates per 100,000 population), (**B**) incidence rate in 2026 (rates per 100,000 population), (**C**) cumulative confirmed cases, and (**D**) cumulative deaths. All maps are represented by color scales, where lighter shades indicate lower rates or values and darker shades indicate higher rates or values. Thematic maps of Mexico illustrating the spatial distribution of cases, deaths, and incidence rates were generated using the map function in Microsoft Excel (version 2026, Microsoft Corporation, Redmond, WA, USA). Figure generated by the authors based on data from SINAVE/DGE/SALUD/Special Epidemiological Surveillance System for measles (accessed on 4 May 2026).

To identify the geographic location and the Mexican states with the highest incidence of measles, cumulative cases, and deaths, we conducted an analysis of the data shown in Figure 1. Figure 1A reflects the incidence rate per 100,000 inhabitants in 2025; the states with the highest incidence were as follows: Chihuahua (113), Jalisco (8), Guerrero (7), Michoacán (5), and Chiapas (4). Figure 1B presents the incidence rate per 100,000 inhabitants for the period from 2026 up to epidemiological week 17. For this timeframe, the most affected states were Jalisco (66), Durango (14), Chiapas (13), Colima (12), and Sonora (10). Figure 1C shows the cumulative number of cases for the start of 2025 though epidemiological week 17 of 2026. The states with the highest number of cases were as follows: Jalisco (6681), Chihuahua (4587), Chiapas (1062), Mexico City (942), and Michoacán (436). Figure 1D represents the cumulative deaths for the entire study period. The states with reported deaths were Chihuahua (21), Jalisco (5), Mexico City (3), Durango (2), Chiapas, Michoacán, Sonora, Guerrero, Sinaloa, and Tlaxcala, with one death each.

**Figure 2:**
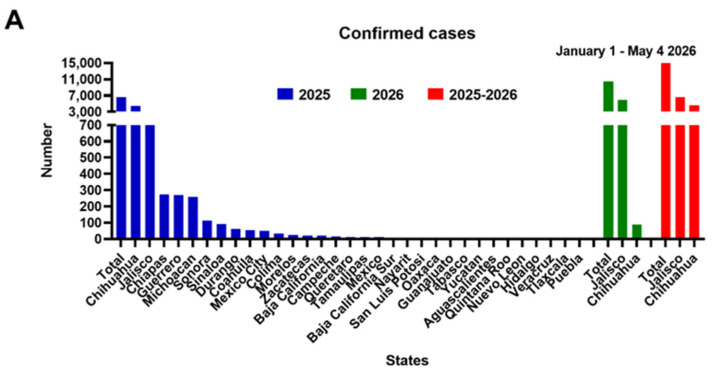

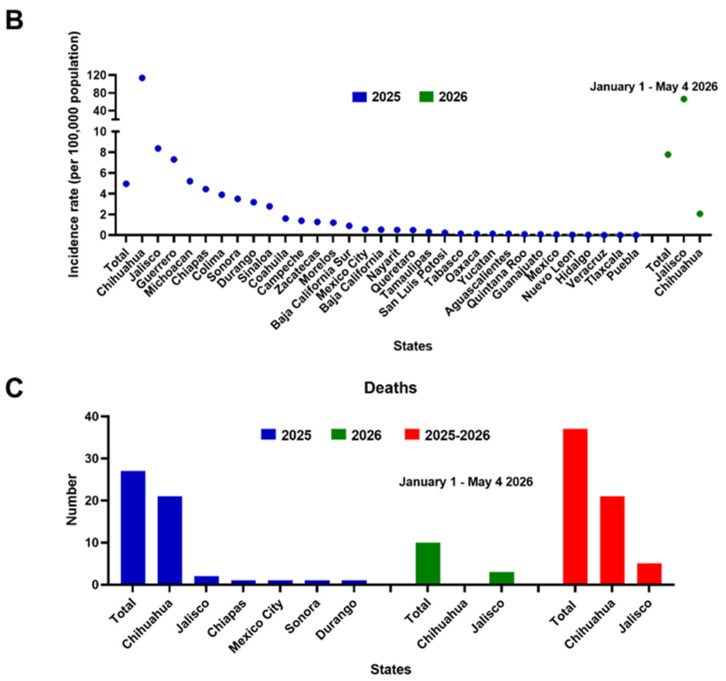
Measles cases, deaths, and incidence in Mexico, 2025–2026. (**A**) Confirmed cases, (**B**) incidence rates per 100,000 population, and (**C**) measles-related deaths. Figure generated by the authors based on data from SINAVE/DGE/SALUD/Special Epidemiological Surveillance System for measles (accessed on 4 May 2026).

The epidemiological dynamics of measles in the country showed a significant transition between the periods analyzed. In 2025 (indicated in blue in Figure 2A), 6608 cases were registered nationwide, with the burden concentrated mainly in five states: Chihuahua (4497), Jalisco (736), Chiapas (273), Guerrero (271), and Michoacán (259). The data for 2026 (represented in green) reveal a substantial distribution of transmission hotspots; while a critical decrease to only 90 cases was reported in Chihuahua, Jalisco experienced an exponential increase, with 5945 confirmed cases. This trend, consolidated by the cumulative figures for both periods (identified in red), demonstrates that the state of Chihuahua showed decisive control over the initial outbreak there. In contrast, Jalisco currently stands as the epicenter of the epidemic in 2026, with 6681 cases reported, contributing to the national total of 17,061 confirmed cases accumulated from 2025 through to epidemiological week 17 of 2026.

According to Figure 2B, the incidence rate per 100,000 inhabitants increased nationally, rising from five in 2025 to eight in 2026. When these data are disaggregated by state, a shift in the focus of the epidemic is revealed. Although the state of Chihuahua registered the highest incidence in 2025, with 113 cases compared to Jalisco’s eight, the trend reversed significantly during the following period. By 2026, Jalisco had consolidated its position of having the highest incidence in the country, reaching 66 cases per 100,000 inhabitants, contrasting with the decrease registered in Chihuahua, where the rate fell to two cases.

Regarding measles-related mortality, in 2025, the state that registered the highest number of deaths was Chihuahua, with 21 deaths, followed by Jalisco, with two. By 2026, a total of 37 deaths had been registered in Mexico, five of which were reported in Jalisco. In contrast, no deaths were reported in Chihuahua during this period, which again reflects this state’s control over the outbreak (Figure 2C).

In light of this context, Jalisco is currently positioned as the epicenter of the measles outbreak in 2026. Therefore, we focus our analysis primarily on this state in order to understand the outbreak as it pertains to this situation.

### 3.2. Epidemiological Features of Measles Outbreaks and Targeted Vaccination Strategies to Close Immunization Coverage Gaps in Jalisco

The response in Jalisco to the measles outbreak was distinguished by a multidimensional approach that combined strengthening the primary vaccination schedule with immediate containment tactics. First, the immunization schedule was optimized by administering two doses of the vaccine (at 12 and 18 months), reinforced by the strategic introduction of the “zero dose” at 6 months of age during the peak season. To interrupt transmission chains in critical settings, school vaccination teams were deployed, and ring vaccination protocols or vaccination blockades were implemented, which prioritized the rapid immunization of close contacts (family, school, work, and neighborhood) of confirmed cases with the aim of establishing an effective immunity barrier. Lastly, these clinical actions were complemented by social mitigation measures, such as the selective closure of classrooms or schools upon detection of positive cases and the implementation of mask use—tools that proved crucial in reducing the spreading of the virus and the safeguarding of public health [4,9,18].

We focused on the state of Jalisco because it is the epicenter of the measles outbreak in Mexico in 2026. This analysis suggests how targeted government interventions, particularly vaccination campaigns, may have contributed to the successful control of the cases in this state. The first reported measles case in Jalisco occurred during epidemiological week 34, which corresponded to August 2025. It involved a two-year-old boy from the municipality of Zapopan who had a complete vaccination schedule, presented with very mild symptoms, and had no history of traveling abroad. Consequently, the origin or source of infection could not be established, and it was therefore considered an isolated case.

Although the first reported cases in Jalisco were identified elsewhere, the municipality of Arandas has been identified as the epicenter of the outbreak in Jalisco. This concentration is attributed to a multifactorial transmission dynamic: first, the mobility of seasonal agricultural workers from Chihuahua, Oaxaca, Veracruz, Chiapas, and Guerrero, from where most of the imported cases originate; second, low vaccination rates; and lastly, the spread of the virus among vulnerable indigenous populations [11].

**Figure 3:**
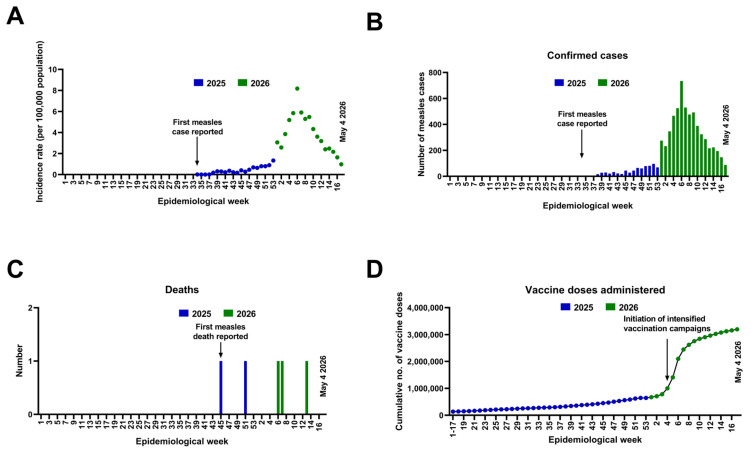
Current epidemiological situation for measles in Jalisco, Mexico (2025–2026). (**A**) Confirmed cases by date of onset of rash. (**B**) Incidence rates per 100,000 people. (**C**) Measles-related deaths, and (**D**) measles vaccine doses administered. An epidemiological week begins on Sunday and ends on Saturday. The year 2025 is divided into 53 epidemiological weeks based on this weekly cycle. Figure prepared by the authors using data from National Epidemiological Surveillance System (SINAVE), specifically from the Special Epidemiological Surveillance System for Measles and febrile Exanthematous Diseases of the General Directorate of Epidemiology (DGE), which is the platform used to register and monitor measles cases. Records of administered measles vaccine doses were also obtained from the specific platform of the National Center for Child and Adolescent Health (CENSIA). The information was processed by the General Directorate of Public Health of the Jalisco Ministry of Health (as of 4 May 2026).

Figure 3 represents the evolution of the 2025–2026 measles outbreak in Jalisco and shows the number of cases since the first reported case, as well as the effect of the mass vaccination campaign in the state. It is noteworthy that the first measles-related death occurred in epidemiological week 45 of 2025, and that the number of cases showed an upward trend, which reached a peak of 735 cases in epidemiological week 6 of 2026. In line with this trend, vaccination campaigns were intensified in epidemiological week 4, as evidenced by the accelerated growth shown in the same figure. The results of this measure are evident in the slowdown in cases, with 492 observed in epidemiological week 9. Lastly, in the last recorded epidemiological week, a substantial decrease in the number of cases was observed, with 88 confirmed cases, which represented an incidence of one per 100,000 inhabitants.

Weekly measles incidence rates differed significantly between Chihuahua and Jalisco throughout the study period (Wilcoxon signed-rank test: W = 1485; Z = −10.80; *p* < 0.001). Weekly case–fatality rates also showed significant differences between the states (W = 4553; Z = −9.26; *p* < 0.001). A weak but statistically significant inverse correlation was observed between the weekly incidence curves (Spearman’s rho = −0.303; *p* = 0.000002), indicating contrasting epidemic trajectories over time. The cumulative incidence rate ratio was 1.48, demonstrating that the overall incidence in Chihuahua was 48% higher than that observed in Jalisco during the study period.

Table 1 describes the population characteristics in the state of Jalisco regarding age group and vaccination status, as well as the unvaccinated cases of measles during the current outbreak. It is worth noting that the 20–49 age group represented 41.96% of cases, and the one to four age group accounted for 12.27%. Another relevant finding is that 63.49% of measles cases were unvaccinated, and almost 20% of those infected were unaware of their vaccination status. For all cases, 8.92% required hospitalization, and among these hospitalized patients, 84.89% were either unvaccinated or had unknown vaccination status. The overall mortality rate was 0.07%.

**Figure 4:**
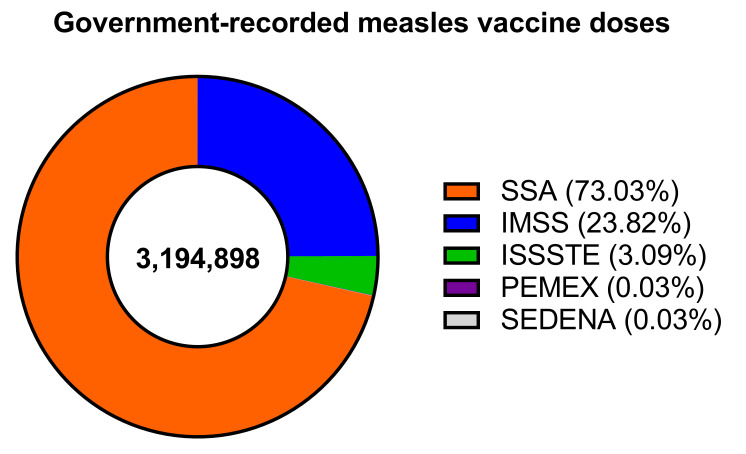
Registered doses of measles vaccines administered by healthcare institutions in Jalisco, Mexico (2025–2026). The chart illustrates the distribution of total vaccine doses administered by the Mexican Social Security Institute (IMSS), the Institute for Social Security and Services for State Workers (ISSSTE), the Mexican Petroleum (PEMEX), the Ministry of National Defense (SEDENA), and the Health Ministry (SSA). Data reflects the collaborative immunization efforts between federal social security providers and public health services within the state during the biennial period. This figure was generated by the authors based on data from registered doses administered and National Center for Child and Adolescent Health CENSIA platform records for measles. The information was processed by the General Directorate of Public Health of the Jalisco Ministry of Health (as of 4 May 2026).

Regarding the distribution of measles vaccine doses administered in Jalisco by government institutions with the support of the private sector, Figure 4 illustrates that the Jalisco Ministry of Health administered the majority of measles vaccine doses for outbreak control, at 2,283,385 out of a total of 3,194,898. The Health Ministry (SSA), the Mexican Social Security Institute (IMSS), and the Institute for Social Security and Services for State Workers (ISSSTE) were the public agencies primarily responsible for the vaccination intensification campaign, which accounted for 99.94% of the doses administered. The Ministry of National Defense (SEDENA) and Mexican Petroleum (PEMEX) administered 1024 and 1005 doses, respectively.

The distribution of measles vaccine doses across age groups is summarized in Table 2. The zero dose, administered exclusively under outbreak conditions, accounted for 1.77% of the total doses administered. Catch-up vaccinations among individuals aged 2–49 years represented the largest proportion of doses administered (72.93%). Targeted vaccination efforts among farmworkers accounted for 1.85% of the total administered doses.

## 4. Discussion

The health stability of the Americas, which in 2016 was declared the first area in the world free of endemic measles, is now at a critical crossroads due to a lack of funding and sustained support [19,20].

The loss of the Americas’ status as the first measles-free zone was due to cases in Venezuela (2018) and later in Brazil (2019). It is important to clarify that this status is lost when the virus circulates endemically (continuous transmission among residents) and persists for over 12 months, distinct from imported cases, which are infections contracted outside the territory [19]. Following a complex response effort, Venezuela regained its measles-free status in 2023, while Brazil, and by extension the entire region, managed to regain the title of a measles-free zone in November 2024 [21].

The recent loss of elimination status in Canada (November 2025), coupled with the resurgence of outbreaks in the United States linked to increased human mobility, has created a regional challenge that places Mexico under unprecedented international scrutiny. This interconnected epidemiological landscape underscores the vulnerability of the Americas to imported cases and local transmission [22].

We observed a substantial difference in the number of measles deaths between Jalisco and Chihuahua. Although both states were national epicenters of the outbreak, Chihuahua disproportionately accounted for the highest number of deaths. This phenomenon requires a multifactorial explanation that considers both the magnitude of the outbreaks and other factors associated with mortality. A systematic analysis identified the main determinants of mortality as a lack of immunization, limited or insufficient access to health services, malnutrition, and delays in medical care [23,24]. Recent evidence on the measles epidemic in Mexico during the 2025–2026 period shows that the state of Chihuahua has reported a significant number of cases and, disproportionately, the majority of deaths. The group with the highest number of deaths included people with high social vulnerability, members of indigenous communities, and unvaccinated individuals [11]. This pattern could suggest that measles mortality is more related to the epidemiological composition of cases than to the absolute number of cases, which may explain the difference in the proportion of deaths between the states of Jalisco and Chihuahua.

In this regard, the variations identified between Jalisco and Chihuahua could be explained by understanding the differences in population immunity. With respect to measles, the variation in state and national vaccination averages could imply increased susceptibility to an outbreak, which has been observed in settings with higher rates of transmission, while recent outbreaks could temporarily reduce susceptibility and improve responses to control outbreaks [25]. In this context, the decrease in cases in Chihuahua in 2026 can be explained by referring to the depletion of susceptible individuals after the 2025 outbreak, coupled with an effective health response, unlike in Jalisco, where a greater proportion of the susceptible population was maintained during that period, which favored the increase in the outbreak in 2026.

As observed in the results of our study, the magnitude of the outbreak in Jalisco was potentially influenced by a lack of immunization, with 63.49% of total cases and 74.83% of hospitalizations occurring in unvaccinated individuals. These data, combined with the high incidence in adults aged 20 to 49 (41.96%), confirm the existence of accumulated pockets of susceptibility that facilitated viral spread and increased the pressure on health services during the 2025–2026 period.

The results of this analysis are consistent with previously reported evidence that the dynamics of measles depend more on the spread of unimmunized people than on routine vaccination coverage [25,26]. In this regard, our results for the state of Jalisco illustrate the progression of the epidemic, with the peak incidence observed at the beginning of 2026, followed by a marked decline in transmission—a trend that coincided with the implementation of intensified immunization campaigns aimed at reducing transmission.

As previously mentioned, measles is preventable with prompt vaccination. Consequently, in order to avoid outbreaks, it is essential to maintain a population vaccination coverage of at least 95% with two doses [27].

It is worth noting that some reports have indicated that imported cases rarely produce sustained transmission if the population is homogeneously immunized. However, if the carrier joins a closed group or community with insufficient vaccination coverage, the disease can spread rapidly [28,29]. This risk is currently materializing with respect to the current border crisis, where it has been documented that the outbreak in Mexico may have been driven by the importation of cases from Texas, which were specifically linked to communities with high mobility and under-immunization in the southern United States [30]. This is consistent with the first cases reported in Mexico, in which a history of previous travel was emphasized, demonstrating that viral reintroduction occurred under conditions favorable for its spread. Based on the results of this study, it is clear that the vulnerability of the affected population was structured by the accumulation of susceptible individuals, the community concentration of unvaccinated individuals, and the barriers to achieving homogeneous vaccination coverage [28,29].

Current evidence shows that, in Mexico, vaccination coverage has consistently fallen below the 95% level needed to maintain elimination status. This phenomenon is not an isolated crisis, but rather a sustained decline. It has been established that, between 2000 and 2018, the rate of completion of the full vaccination schedule for children under five remained below 90% [31]. Although recent international reports have placed coverage of the first dose between 80 and 89% [21,32], national data from the 2021–2023 National Survey of Health and Nutrition (ENSANUT) reveal an even more critical picture: coverage of the first dose of MMR in children under five was only 71.3%, with just 32.4% of children completing the full vaccination schedule by 24 months. This weakening trend has been exacerbated by the current outbreak, with reports indicating that first-dose coverage has fallen to 65% [30]. This scenario is further challenged by constraints in health system capacity, with predictive models estimating that by the end of 2026, immunization coverage may reach only 85.41%. This deficit is largely attributed to the logistical demands following the 2025 outbreak [33]. These figures not only explain the historical accumulation of susceptible individuals but also underscore a systemic decline in vaccination opportunities, which is consistent with the resurgence of outbreaks observed in Jalisco and the rest of the country during the 2025–2026 period [11].

Low vaccination coverage is influenced by a combination of vaccine hesitancy, widespread misinformation, and declining trust in health institutions and pharmaceutical companies. False claims regarding vaccine safety, particularly those disseminated through social media and informal communication networks, can amplify concerns about adverse effects and reduce confidence in immunization programs. In addition, limited health literacy and inadequate communication with trusted healthcare professionals may further delay or discourage vaccine uptake, contributing to the re-emergence of vaccine-preventable diseases such as measles [34,35].

Recent studies on measles have revealed accumulated shortcomings in vaccine administration. A 19-year study, spanning from 2006 to 2024, showed that only 69 million doses were administered out of the 91.6 million doses required. This represents a deficit of approximately 25% for the target population [32]. A study focused on vaccine inequity in Mexico reported an increase in the proportion of children without the MMR/MR vaccine, rising from 10.2% in 2012 to 29.1% in 2021, thus demonstrating a significant decline in coverage [36]. This evidence is consistent with the current resurgence of outbreaks in the country, which is related to suboptimal vaccination coverage.

Given this accumulated deficit, the scientific literature suggests that outbreak control requires more than simply increasing vaccinations, but rather the implementation of a territorially focused strategy. This involves the use of mobile vaccination teams and ring vaccination campaigns in critical micro-areas, which are reinforced by vaccine literacy programs that utilize trusted messengers to combat misinformation and close operational gaps [37]. Ring vaccination is an outbreak containment strategy that involves the prompt immunization of individuals who have been in close contact with confirmed cases, as well as their secondary contacts, to establish a surrounding layer of immune individuals that limits further spread of the infection [38].

Regarding the strategies implemented in the state of Jalisco, it is important to highlight that they are consistent with international recommendations for the management and control of measles outbreaks. To this end, the ring vaccination system has stood out as an effective intervention for interrupting transmission in environments with a high level of contagion [18]. Another strategy implemented is the administration of the zero dose in the six-to-eleven-month age range, which is a recommendation from international organizations to protect vulnerable groups that are not within the established age range for vaccination [39]. This reflects an alignment in the response developed in Jalisco with international strategies for containment in high-transmission scenarios.

Regarding the transmission dynamics observed in Jalisco, it is worth noting that this is consistent with previous evidence indicating that population mobility plays a central role in the spread of measles, since multiple studies have documented that migratory flows and seasonal work facilitate the introduction and dissemination of the virus in communities with heterogeneity, with respect to the level of vaccination coverage [40,41].

Among the most notable findings is the predominance of cases in adults aged 20 to 49 (41.96% of the total), which suggests that a significant proportion of this disease burden could have been avoided. This reflects previous and accumulated inconsistencies in immunization coverage, thereby leading to heterogeneity in coverage in previous years. These results are consistent with previous evidence in elimination or post-elimination scenarios in which outbreaks are no longer primarily affecting infants, but instead are impacting young adult and adult populations [42,43]. Moreover, local evidence from the population in Jalisco has confirmed a high density of susceptible adults in this same age group due to historical vaccination coverage gaps [44]. Similarly, recent international data from southern Italy highlight a substantial immunity coverage gap among young adults (18–34 years), where zero-dose susceptibility remains high despite previous vaccination efforts, thereby underscoring a decline in vaccine-induced immunity over time [45]. Through measles outbreaks, gaps in vaccination coverage have been identified in specific subpopulations, even in contacts with reports of high coverage [46]. This suggests that the dynamics of the outbreak in Jalisco affect not only children but also adults who are susceptible, owing to a lack of timely vaccination coverage, which in turn increases transmission.

Our findings are consistent with recent data from the 2024 measles epidemic in Romania, where adults accounted for a substantial proportion of hospitalized cases. In our study, individuals aged 20–49 years made up the largest cohort of confirmed cases (41.96%). Notably, Vâță et al. (2026) reported markedly greater systemic severity in adult patients compared to pediatric cohorts, characterized by higher hepatic involvement (ALT elevation in 63.1% vs. 34.2%) and increased frequency of thrombocytopenia [47]. These international data highlight the importance of conducting future clinical studies in our population to systematically monitor and characterize these specific biochemical and hematological complications. Collectively, these observations suggest a shifting epidemiological trend toward young and middle-aged adults in contemporary outbreaks, emphasizing the need to improve vaccination coverage and reinforce targeted surveillance strategies.

The high proportion of unvaccinated cases, as well as hospitalization among individuals without a history of vaccination or with unknown vaccination status, is consistent with previous reports showing that the vaccinated population has a lower risk of hospitalization, while morbidity and mortality are increased in the unvaccinated population [48]. Recent reports have suggested that vaccination reduces both the risk of infection and the likelihood of severe disease and complications [1,23]. In this regard, the data reported in Jalisco strengthen the assessment that the severity of the outbreak is highly linked to the lack of vaccine coverage in the affected population.

Another epidemiologically relevant group is children under five years of age, since there are reports that infants and young children are often highly susceptible during outbreaks due to a lack of maternal antibodies and because they are younger than the programmatic age for complete vaccination [1]. An additional factor is that this age group overlaps with the cohort of children who did not receive their basic schedules of immunization owing to the disruption of health services and confinement during the COVID-19 pandemic, which has exacerbated the accumulation of susceptible individuals globally [49].

In addition to primary susceptibility, it is critical to note that MeV paradoxically induces transient immunosuppression, or immune amnesia. This process involves the depletion of memory lymphocytes, significantly increasing vulnerability to secondary infections for months following the acute phase [5,6]. This immune impairment likely contributed to the 8.92% hospitalization rate observed in Jalisco, as secondary complications are frequently more severe in under-immunized populations with virus-induced suppression [1,24].

The high proportions of doses administered through catch-up vaccination programs in the 20–49 age group further demonstrate these gaps in partial or complete immunization coverage, which act as a determining factor for measles re-emergence [43,45,50].

Consequently, the outbreak dynamics observed in Jalisco affect not only children but also susceptible adults due to a lack of timely vaccination coverage. Within this framework, implementing recovery or rescue vaccination strategies in these specific groups is highly effective for interrupting transmission and reducing the susceptible pool that keeps the outbreak active [44,45,51,52].

In this regard, data from our study support this institutional effort at the state level, with 3,194,898 total vaccine doses administered, of which 49.19% were given to adults aged 20 to 49 years. These data clearly indicate that the distribution of doses in Jalisco is in accordance with a strategy to reduce the historical immunization gap and also includes specific interventions such as the zero dose for infants and the protection of strategic groups such as healthcare workers and farm laborers.

Lastly, Jalisco positioned itself as a global health security hub in the lead-up to the 2026 International Federation of Association Football (FIFA) World Cup, which will be held in June and July. The magnitude of the anticipated large-scale population movement into Guadalajara, coupled with the state capital, and demands that local interventions meet international standards mean that vaccination coverage will be prioritized, with expectations exceeding 95%. Mass vaccination campaigns and containment strategies have already facilitated a significant reduction in measles cases in the state, thereby mitigating the risk of spread stemming from uneven immunization coverage [11]. This operational success not only encompasses the current outbreak [11] but will also protect the region from the upcoming sporting event, acting as a catalyst for viral export or reintroduction. This resilience is based on robust inter-institutional collaboration, with the SSA leading the effort by administering 71.49% of the doses, supported by the strategic participation of IMSS (24.94%) and ISSSTE (3.52%). This synergy was recently bolstered by Mexico and Jalisco’s active participation in the 24th Vaccination Week in the Americas and the 15th World Immunization Week, which began on 25 April 2026. While these initiatives traditionally span a single week, the vaccination campaign in Jalisco and throughout the country has recently been extended to cover the entire month of May. During this period, vaccination efforts have intensified nationwide to address multiple vaccine-preventable diseases, with a strategic focus on measles. This timely extension serves as a critical tool to close remaining immunity coverage gaps just days before the global influx of visitors. This synergy guarantees epidemiological stability during the World Cup and underscores the capacity of the state health system to respond to global threats.

Several limitations of this study should be considered. First, the descriptive, ecological time-series design does not allow for definitive causal inference. Although the decline in incidence coincides with increased vaccination, other factors such as natural depletion, susceptibility, regression to the mean, or seasonal transmission fluctuations may also have contributed. Without a formal interrupted time-series regression framework to control these cofounders, our findings reflect temporal associations rather than causal attributions. Second, the retrospective nature of the data may introduce information bias related to self-reported vaccination status and incomplete records. Notably, vaccination status was unknown in 19.57% of cases, potentially leading to non-differential misclassification of the primary exposure. This type of bias would be expected to attenuate the observed association between vaccination status and hospitalization, suggesting that the protective effect of vaccination may be underestimated. Third, our study relied on passive surveillance data from SINAVE, which may be subject to underreporting and selection bias, with a likely overrepresentation of symptomatic and hospitalized cases. Consequently, estimates of hospitalization, age distribution, vaccination status, and case fatality may need to be interpreted cautiously, as they may not fully reflect the characteristics of all measles infections in the population. Similar limitations have been described for other national passive surveillance systems, including a recent evaluation conducted in Lebanon, which reported substantial variability in the completeness and timeliness of case investigations [53]. Fourth, the lack of molecular sequencing limits the characterization of transmission chains. Finally, projections for 2026 rely on models subject to logistical or social shifts. Nevertheless, the consistent patterns observed across multiple sources and timeframes reinforce the robustness of our core conclusions.

## 5. Concluding Remarks

Despite control efforts, measles remains a major threat to global public health, especially in the face of large-scale events like the 2026 FIFA World Cup. The convergence of millions of international visitors in Jalisco presents an unprecedented epidemiological challenge, as large-scale global mobility tests local immunity coverage. In this scenario, robust immunization programs and active surveillance are not only preventive measures but critical pillars for avoiding an outbreak of international magnitude. Therefore, achieving optimal vaccination rates is essential for consolidating herd immunity in the face of this large-scale movement of people and for safeguarding public health.

## Figures and Tables

**Table 1:** Characteristics and vaccination status of confirmed measles cases during the outbreak: Jalisco, Mexico (epidemiological week 34, 2025–epidemiological week 17, 2026).

Characteristic	No. (%)
Total	6681 (100%)
Sex	
Female	3202 (47.92%)
Male	3479 (52.07%)
Age group	
Median (range)	18
<1 year	433 (6.48%)
1–4 years	820 (12.27%)
5–9 years	1010 (15.11%)
10–19 years	1412 (2113%)
20–49 years	2804 (41.96%)
>50 years	202 (3.02%)
Vaccination status (cases)	
Vaccinated, 2 doses (or more)	267 (3.99%)
Vaccinated, 1 dose	864 (12.93%)
Unvaccinated	4242 (63.49%)
Unknown	1308 (19.57%)
Outcome	
Hospitalized	596 (8.92%)
Died	5 (0.07%)
Vaccination status of hospitalized patients	
Vaccinated, 2 doses (or more)	15 (2.51%)
Vaccinated, 1 dose	75 (12.58%)
Unvaccinated	446 (74.83%)
Unknown	60 (10.06%)

**Table 2:** Measles vaccine doses administered during the outbreak: Jalisco, Mexico (epidemiological week 1, 2025–epidemiological week 17, 2026).

Characteristic	No. (%)
Total	3,194,898 (100%)
Group	
(6–11 months)	56,576 (1.77%)
First dose (12 months)	198,474 (6.21%)
Second dose (18 months)	215,929 (6.75%)
Second dose (6 years)	234,133 (7.32%)
Catch-up doses (2–9 years)	191,670 (5.99%)
Catch-up doses (10–19 years)	557,041 (17.43%)
Catch-up doses (20–39 years)	1,094,609 (34.26%)
Catch-up doses (40–49 years)	477,112 (14.93%)
Healthcare workers	77,554 (2.42%)
Educational staff	32,509 (1.01%)
Farmworkers	59,291 (1.85%)

## Data Availability

The data presented in this study are available on request from the corresponding author.
